# Enhancing Hospital Efficiency and Patient Care: Real-Time Tracking and Data-Driven Dispatch in Patient Transport

**DOI:** 10.3390/s24124020

**Published:** 2024-06-20

**Authors:** Su-Wen Huang, Shyue-Yow Chiou, Rung-Ching Chen, Chayanon Sub-r-pa

**Affiliations:** 1 Taichung Veterans General Hospital, Taichung 40705, Taiwan; dale33663366@gmail.com (S.-W.H.); yow0819@vghtc.gov.tw (S.-Y.C.); 2 Department of Information Management, Chaoyang University of Technology, Taichung 413310, Taiwan

**Keywords:** inpatient transportation, data-driven dispatch, process optimization, healthcare technology

## Abstract

Inefficient patient transport in hospitals often leads to delays, overworked staff, and suboptimal resource utilization, ultimately impacting patient care. Existing dispatch management algorithms are often evaluated in simulation environments, raising concerns about their real-world applicability. This study presents a real-world experiment that bridges the gap between theoretical dispatch algorithms and real-world implementation. It applies process capability analysis at Taichung Veterans General Hospital in Taichung, Taiwan, and utilizes IoT for real-time tracking of staff and medical devices to address challenges associated with manual dispatch processes. Experimental data collected from the hospital underwent statistical evaluation between January 2021 and December 2021. The results of our experiment, which compared the use of traditional dispatch methods with the Beacon dispatch method, found that traditional dispatch had an overtime delay of 41.0%; in comparison, the Beacon dispatch method had an overtime delay of 26.5%. These findings demonstrate the transformative potential of this solution for not only hospital operations but also for improving service quality across the healthcare industry in the context of smart hospitals.

## 1. Introduction

**Figure 1:**
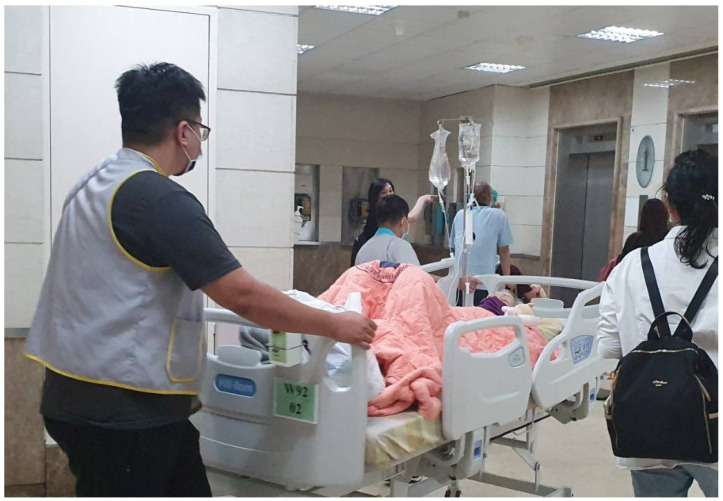
Nurses wait in line to access the facility during the patient dispatch process.

Efficient patient flow is critical for optimal hospital operations, impacting patient satisfaction, resource utilization, and overall costs; however, many hospitals, including Taichung Veterans General Hospital in Taiwan, need help with inpatient transportation. These challenges often stem from budget constraints and staffing shortages, further exacerbated by systemic issues within the Taiwanese healthcare system [1,2,3], such as limited government oversight and commercial pressures. Inefficient, manual processes for inpatient transportation can lead to bottlenecks, delays, and increased staff workload, hindering timely care delivery (example in Figure 1) and contributing to patient dissatisfaction.

The current method for assigning dispatch work at Taichung Veterans General Hospital is inefficient and time-consuming. It relies on phone calls or waiting for orderlies to return to the service center before new tasks are assigned. This delay in task allocation increases physical strain on the orderlies and impacts patient care.

In 2019, Taichung Veterans General Hospital took proactive steps to address challenges by implementing an Internet of Things (IoT) device for a real-time positioning system called “Beacon”. The new data were integrated into the existing system, which included a new algorithm for dispatch management called the “Beacon dispatch system”. The new system leveraged advanced information technology (IT) [4,5,6,7,8] and the IoT [8,9,10,11] to develop and implement a data-driven dispatch algorithm that optimizes the use of Beacon. This algorithm integrates real-time tracking of patients, staff, and equipment with hospital information systems, empowering dispatchers to make informed decisions, automate processes, optimize resource allocation, and predict potential bottlenecks.

Recognizing the potential risks of abruptly implementing new systems in a complex healthcare environment, we adopted a phased approach at Taichung Veterans General Hospital. We collected data for analysis covering January 2021 to December 2021, prioritizing patient safety and minimizing disruptions to care. This phased implementation involved:

1. Testing Phase—the Beacon dispatch system underwent thorough testing in a controlled environment, and the staff underwent training to comprehend the new workflow. Throughout this phase, personnel received operational training to ensure familiarity with the system and to prevent disruptions to existing workflows;
2. Limited Shipment Phase—the new system was gradually applied to emergency cases where the service center received a dispatch request for a specific staff member who could reach the starting point within 15 min;
3. Full-Level Phase—the Beacon algorithm was fully implemented across the entire hospital, encompassing all case levels (emergency, non-urgent, scheduled). All personnel received the necessary training for automatic dispatch, regardless of their department or role.

We employed process capability analysis and simulation to ensure our interventions were well-informed and tailored to the hospital’s unique needs [12,13,14,15]. This systematic, data-driven approach [16,17,18,19] allowed us to meticulously map current workflows and quantify system performance, facilitating targeted improvements while minimizing disruptions.

Through statistical evaluation of the new system’s real-world performance, we aim to demonstrate its impact on key operational metrics, including pickup delay (patient waiting times), dispatch delay, and dispatch delay count. This study provides valuable insights into integrating IT, IoT, and process optimization methodologies to revolutionize inpatient transportation, enhancing patient care and operational efficiency within a complex hospital environment. Importantly, our approach underscores the significance of balancing innovation with patient safety in healthcare settings.

Our research makes several key contributions to the field:

- Real-World Implementation—we demonstrate the successful application of process capability analysis, simulation, and IoT technology to address inpatient dispatch challenges in a real hospital setting;
- Data-Driven Dispatching—we gathered and analyzed data from our experiment to enhance hospitals’ resource allocation through technology-driven dispatch algorithms for increased efficiency and future development;
- Patient-Centric Approach—our study prioritizes patient safety and emphasizes the importance of minimizing disruptions to care while implementing new processes with a phased implementation approach.

The rest of this paper is structured as follows: Section 2 details related work, including process capability analysis, which is the foundation for our algorithm; Section 3 outlines our methodology; Section 4 presents experimental results and analysis; and Section 5 concludes this study.

## 2. Related Work

### 2.1. Patient Flow Optimization and Inpatient Transportation

Centralized command centers are essential for effectively coordinating the transportation of patients and medical devices within hospitals. These centers have historically relied on telephone communication to orchestrate the allocation of staff and resources; however, this traditional method has presented several challenges, such as communication delays, the potential for miscommunication, and difficulty tracking the status of patient flow in real-time.

Studies on patient flow optimization and inpatient transportation have focused on improving hospital efficiency and patient care [20,21]. According to [21], research was carried out on a large hospital’s patient transportation system to propose a simulation-optimization solution. The aim was to enhance efficiency and reduce demand completion times. The study emphasized the importance of data analysis in understanding the complexities of patient transportation systems. It also suggested that hospitals can enhance performance by identifying bottlenecks and inefficiencies through targeted solutions. Furthermore, the research found that many hospitals encounter similar challenges in patient transportation.

Refs. [1,2] highlight the detrimental impact of transportation delays on overall patient flow and explore strategies for optimizing resource allocation, such as staff and vehicles. Simulation models frequently test different transportation approaches and identify optimal solutions without disrupting real-world hospital operations.

The reliance on individual phone calls to ascertain staff availability and assign tasks proved time-consuming, particularly during peak hours or in large hospitals. Real-time monitoring was limited, relying on periodic check-ins that might not reflect the dynamic nature of hospital operations. Furthermore, manual documentation via phone calls increased the risk of errors and inconsistencies. As hospitals expanded and patient volumes grew, this approach became increasingly cumbersome and inefficient.

Hospitals are increasingly adopting modern technologies such as hospital management systems, real-time location systems [22,23] (e.g., our proposed Beacon), smartphones, and integrated communication platforms to address the limitations of telephone-based communication. These advancements provide real-time visibility into staff availability and location, streamline communication, automate documentation, and ultimately enhance patient dispatch systems’ overall efficiency and responsiveness.

### 2.2. Real-Time Tracking and the Internet of Things (IoT) in Healthcare

The Internet of Things (IoT) [8,9,10,11] is revolutionizing healthcare by extending beyond mere asset tracking. IoT devices, including sensors, actuators, and other networked gadgets, are now integrated into various aspects of patient care. For example, smart beds equipped with sensors can continuously monitor vital signs, movement, and sleep patterns, providing valuable data for personalized treatment plans and early detection of potential issues.

Bluetooth Low Energy [23] (BLE) technology is crucial in healthcare facilities by enabling precise, real-time location tracking of medical equipment and patients. This technology streamlines workflow processes, improving efficiency and patient safety. By leveraging BLE, healthcare providers can ensure timely access to necessary medical equipment and personnel, ultimately enhancing the overall quality of patient care.

Integrating IoT with the Artificial Intelligence of Things (AIoT) [24,25,26] further expands the possibilities. AI algorithms can analyze the vast amounts of data generated by IoT devices, revealing patterns and trends that can inform decision-making. This can lead to insights such as optimal equipment storage locations, predictive maintenance to prevent failures, and personalized patient care recommendations.

The healthcare industry is currently undergoing a significant transformation due to the integration of IoT and AIoT. For example, wearable IoT devices [11] are crucial in continuously monitoring patients’ vital signs remotely and in real-time locations, allowing for timely interventions during emergencies. Furthermore, IoT-powered medication dispensing systems are improving patient care by ensuring precise and timely delivery of medications, thereby reducing errors and enhancing overall patient safety.

While the potential of IoT in healthcare is vast, challenges such as data security, privacy, system integration, and data management must be addressed to ensure the successful and ethical implementation of these technologies. Furthermore, rigorous testing and validation methodologies are essential to ensure that these technologies benefit patients and perform within a secure and reliable framework. This includes comprehensive testing in controlled environments, phased rollouts to monitor real-world performance, and continuous evaluation to identify and address potential issues. By prioritizing patient safety and data security alongside technological innovation, healthcare providers can harness the full potential of IoT to revolutionize patient care while upholding the highest ethical standards.

### 2.3. Simulation Modeling in Healthcare

While simulation [12,13,14,15] has emerged as a valuable tool for testing different patient flow management approaches without risking operational disruptions or compromising patient safety, concerns remain about its ability to fully capture the complexities and nuances of real-world hospital environments. This includes factors like unpredictable patient arrivals, staff behavior variations, and the dynamic nature of healthcare processes.

For instance, a study [12] focusing on a regional hospital’s emergency department (ED) utilized process capability analysis and simulation to assess its ability to meet service-level goals. By analyzing existing processes and resources and simulating potential improvements, the study identified the most cost-effective solution: converting a part-time doctor to full-time, reducing wait times, and enhancing patient flow. However, the study acknowledged the limitations of the simulation model in fully capturing the intricacies of real-world ED operations.

The research on Simulation Modeling in Healthcare [26] emphasizes the multifaceted challenges of integrating these simulation models into current healthcare workflows. Notably, technical incompatibilities stemming from varying systems, data format issues resulting from disparate information structures, and resistance to change within healthcare organizations present significant barriers to successful integration efforts. Overcoming these barriers often demands extensive time, resources, and organizational change management investments to seamlessly incorporate simulation models into healthcare operations.

Our study aims to leverage a simulation model [12] within a real-world hospital setting to address this research gap. By meticulously calibrating the model with actual hospital data and incorporating real-time feedback, we aim to enhance its fidelity and ensure it accurately reflects the complexities of the hospital environment. This approach will allow us to confidently evaluate the impact of various process enhancements on patient flow and resource allocation, ultimately seeking to optimize hospital operations and elevate the quality of patient care.

## 3. Methodology and Experimental Setup

In 2019, the Taichung Veterans General Hospital in Taichung, Taiwan, implemented a real-time location dispatch system called Beacon. This system uses IoT to track the movement of staff, patients, and medical equipment. The aim was to create an intelligent dispatch system that could automatically assign the most suitable and closest available staff member to handle tasks such as transporting patients or medical devices. This was expected to improve the efficiency of healthcare-related transport services by reducing travel distances and streamlining the transport process.

### 3.1. Data Collection

**Figure 2:**
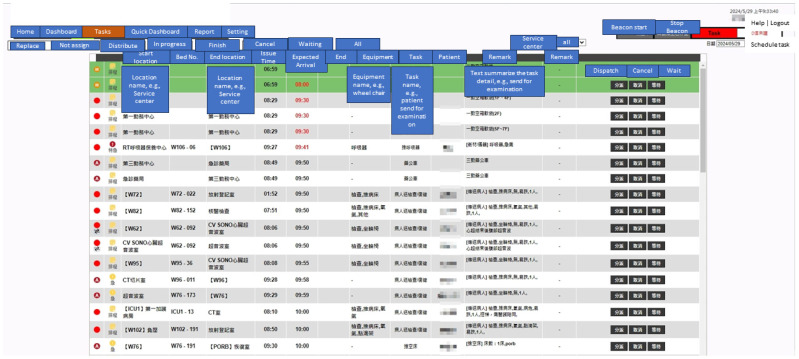
Software interface for managing patient transport in a hospital, with tasks assigned manually by the command center.

Figure 2 shows the software interface of the new integrated system that supports both traditional and Beacon dispatch systems. This study collected data from the system covering January to December 2021. The focus was on daytime (8:00 a.m. to 5:00 p.m.) and nighttime (6:00 p.m. to 10:00 p.m.) to accommodate staffing levels and task volume variations. The collected data include detailed information for each dispatch task. To analyze the new system’s impact, we looked at the date and time of dispatch start, expected dispatch finish, dispatch time limit (acceptable delay), and dispatch finish time. The expected dispatch finish time is when it takes to travel between the originating and destination points. The urgency level determines the dispatch time limit, which will be explained further in Section 3.3.

Equations (1)–(3) represent the fundamental calculation for important values required in evaluation and analysis. Dispatch time refers to the time taken for each dispatch, pickup delay is the time that staff spends traveling to the initial dispatch point, and dispatch delay is the time staff takes after picking up a patient or device to deliver it to the destination.

(1) Dispatch Time=Dispatch finish−Dispatch start

(2) Pickup delay=Dispatch finish−Expect dispatcht finish

(3) Dispatch delay=Dispatch time−Pickup delay

This detailed and extensive dataset has allowed us to conduct an in-depth analysis of the system’s performance and effectiveness. Through this analysis, we have gained valuable insights into crucial metrics such as dispatch delay, the time from dispatch to task start, and the overall efficiency of the transport process. This comprehensive examination has allowed us to gain a nuanced understanding of the system’s operations and identify areas for potential improvement.

### 3.2. Beacon Installation and Integration

The hospital placed 350 Bluetooth Beacon devices across 523 location IDs. Each location ID represented a specific area within the hospital, such as a patient room, nursing station, operating room, or waiting area. Due to the proximity of certain areas and variations in staff and patient density, some Beacons were assigned to cover multiple adjacent location IDs, optimizing the system’s efficiency.

The Beacon system was seamlessly integrated with the hospital’s existing dispatch system. Upon receiving a transport request, the intelligent system rapidly (within approximately 1.1 s) identified the nearest available staff member through their Beacon device’s unique identifier (UUID). The system employed conditional logic to ensure optimal task allocation, considering factors such as the staff member’s current availability, qualifications for the specific task, and proximity to the pick-up location relative to the task’s urgency.

### 3.3. Dispatch Strategy

**Figure 3:**
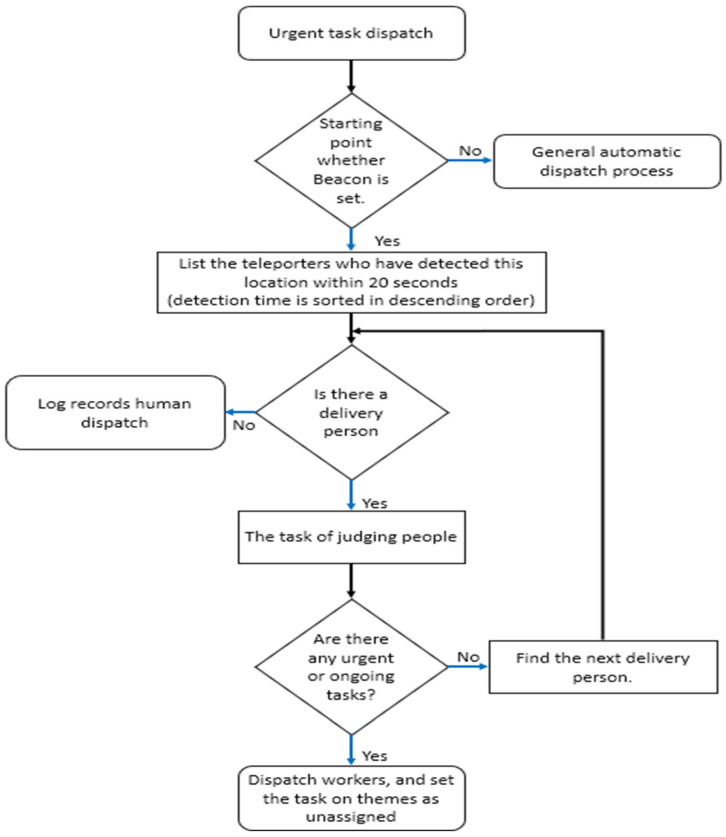
Overview of Beacon dispatch system workflow.

Our experiment utilizes the dispatch strategy of process capability analysis and Simulation [9]. We have modified and implemented this strategy to align with our experiment’s objective and have presented it visually in Figure 3. This process prioritizes tasks based on two critical factors: urgency level and pickup time, which are estimated by the distance between staff and the dispatch point. Urgency levels are categorized as follows:

- Emergency—requires immediate response for life-threatening conditions or situations where a patient’s condition could rapidly deteriorate. Examples include cardiac arrest, severe trauma, or difficulty breathing;
- Urgent—requires a response within 15 min for conditions that need prompt attention but are not immediately life-threatening. Examples include severe pain, high fever, or suspected broken bones;
- Routine or Scheduled—this category encompasses scheduled tasks or non-urgent requests that can be completed within 30 or 60 min. Examples include patient transfers between units, delivery of routine lab specimens, or transportation of medical equipment.

The pickup time for transport staff to arrive at the patient or item pick-up location is determined based on the assigned urgency level. For emergency tasks, the pickup time is immediate. For urgent tasks, the pickup time is set to 15 min. Routine tasks can be completed within a pickup time window of 30 to 60 min, taking into account the specific circumstances of the request, such as location, traffic, and other relevant factors.

The Beacon dispatch system operates with a 10-s scanning interval to identify available personnel. During each scan, the system prioritizes tasks of pickup times within the next 3 min. Within this window, tasks are further sorted by urgency level, ensuring that the most time-sensitive requests are addressed first.

The system initially searches for available personnel on the same floor as the pickup location to optimize response times. If no suitable staff members are found, the search expands to adjacent floors, prioritizing those closest to the pickup location based on estimated travel time. This travel time calculation takes into account factors like distance and typical walking speed to the pickup location.

When staff members are unavailable within the same building, the Beacon system can extend the search to adjacent buildings, ensuring that urgent and emergency requests are always promptly fulfilled.

The choice of dispatch method can significantly impact expected delays due to variations in search and assignment methods. The hospital staff available for dispatch tasks can be denoted as the following:

(4) $$S=\{S_{1},\;S_{2},\;S_{3},\;\ldots \;S_{n}\}$$

where *S* represents staff and n is the number of staff in the work shift. Each *S_i_* has properties like availability and location.

(5) $$S_{i}\in {Location}_{j}$$

where *Location_j_* is the coordination of location (*x_j_*, *y_j_*, *z_j_*). In traditional dispatch, a staff member *Si* is randomly selected for each task (*Ti*). This can lead to errors, rejections, and the need to repeat the search, resulting in a worst-case time complexity of *O*(*n*).

On the other hand, the Beacon dispatch system sorts the list of available staff based on relevant criteria (e.g., proximity, availability) before attempting to assign the task. While Beacon still uses a sequential search with a worst-case time complexity of *O*(*n*)*,* its sorted list allows for potential early termination, improving average-case performance. Furthermore, Beacon aims to achieve high assignment success rates and prioritizes assigning tasks to staff located near the task’s starting point.

### 3.4. Experimental Setup

**Figure 4:**
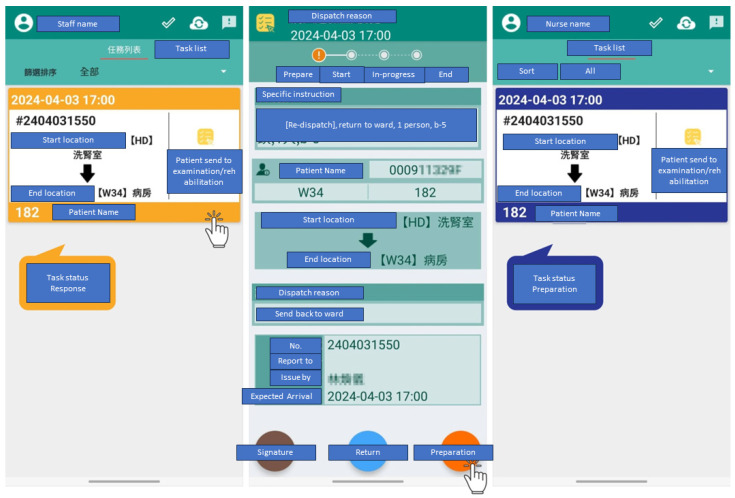
Application used to receive and update the status of dispatch tasks.

The experiments were conducted in a real-world hospital environment. The new Beacon dispatch system is integrated to automatically distribute tasks to existing mobile applications, as shown in the example application in Figure 4. The hospital’s transport staff were classified into two distinct groups: distributed dispatch staff (assigned to specific wards or departments) and centralized dispatch staff (working in shifts across service centers). The task’s urgency dictated the transport staff’s response time: immediate response for emergencies, within 15 min for urgent cases, and 30 or 60 min for routine tasks.

One limitation of this real-world experiment is the variability in case volume due to external factors. Consistent numbers across periods are not guaranteed because the number of cases cannot be controlled experimentally. Instead, we focus on analyzing percentage changes in key metrics to assess the impact of the proposed system. To manage potential risks and ensure a smooth transition, implementing the Beacon dispatch system was divided into three phases.

#### 3.4.1. Testing Phase

Primarily relying on traditional dispatch methods, a small percentage (<20%) of routine tasks were assigned to the Beacon system to assess its functionality and familiarize staff with its operation.

#### 3.4.2. Limited Shipment Phase

The majority of tasks continued to be managed through traditional dispatch; however, all urgent and emergency cases were assigned to the Beacon dispatch system, allowing for a gradual increase in workload as confidence in its performance grew.

#### 3.4.3. Full-Level Phase

All transport cases (emergency, urgent, and routine) were handled exclusively by the Beacon system, fully replacing the traditional dispatch method. Since this phase has a highly disabling effect on the traditional dispatch system, we experimented with this phase for a very limited time.

### 3.5. Evaluation

The Beacon intelligent dispatch system’s performance underwent comprehensive evaluation before and after its implementation. Key metrics were utilized to assess its effectiveness, including:

- Dispatch delay count—the number of dispatch cases completed after the expected dispatch time;
- Average dispatch time—the average time it takes for a task to be dispatched from creation to completion;
- Average Pickup delay—the average time selected staff travel to the dispatch starting point;
- Average Dispatch delay—the average time it takes for staff to dispatch from the starting point to completion.

## 4. Results and Discussion

Implementing the Beacon intelligent dispatch system at Taichung Veterans General Hospital led to significant improvements in various operational metrics. These improvements were consistently observed throughout the three phases of implementation: testing, limited shipment, and full-level. In contrast, the traditional method relied on telephone communication and manual management.

### 4.1. Testing Phase

In this phase, we were testing and training our staff to use the Beacon dispatch system in brief. Once the system was in place, we provided the required training for personnel to ensure the smooth operation of existing transportation services. Following online testing and adjustments, we present the traditional dispatch and Beacon dispatch systems’ statistical analyses—see Table 1 for the daytime shifts and Table 2 for the nighttime shifts.

The outcomes obtained during the testing phase of the Beacon dispatch system indicate that implementing the system resulted in lower delays and average pickup times compared to the traditional method. We applied the Beacon dispatch system to a small sample (±20% of all dispatch cases) to ensure accuracy and overall service quality during testing. During the daytime shift in January, the delay rate of the traditional method was 36.5%, while the new method only had a delay rate of 23.1%.

The average dispatch time of the Beacon dispatch system is only 4 min and 43 s, while the traditional method takes 7 min and 14 s. This trend was consistent in February, indicating that the Beacon dispatch system could improve dispatching by 34% in the daytime shift. Reducing dispatch times can also lower the workforce needed to operate the system. These results suggest that the new system is an effective solution to enhance the overall efficiency of the dispatch process.

However, Table 2 indicates that our new system does not improve dispatch tasks during nighttime shifts. Although the delay rate remains similar to the traditional method, the dispatch time is higher. We cannot conclude whether the negative effect stems from the system or staff understanding. To gain more insight, the experiment was continued into the next phase.

It is important to note that our system’s test phase only covers a portion of the events that can occur in a hospital. For example, the experiment may not have considered situations during rush hour, emergencies, or sudden changes. We continued experimenting with our system in both limited shipment and full-level phases to gain a more comprehensive understanding.

### 4.2. Limited Shipment Phase

In this phase, the Beacon dispatch system transitioned from the previous phase to focusing only on emergency tasks. The traditional methods were mainly used for urgent and routine tasks. The experiment of this transition occurred from March to August, and the results are shown in Table 3 and Table 4.

The transition of the experiment slowly began in March and April. Initially, the two-month trend of statistical evaluation resembled the testing phase. However, from June, the number of dispatch cases handled by the Beacon dispatch system dropped significantly, indicating that our experiments encompassed general and urgent cases, managed by the traditional method, and the Beacon dispatch system handled all emergency cases.

According to Table 3, the Beacon dispatch system improves dispatch time by 78% and pickup time by 32%. The average dispatch process takes less than 15 min, meeting the requirement for emergency dispatches.

Table 4 indicates a low number of dispatch cases in the Beacon dispatch system during the night shift of the limited shipment phase from March to August. Starting in June, the number of dispatch cases increased during the day shift, raising concerns for the hospital about the overall impact of the experiment on the service. As a result, the Beacon dispatch system experiment was scaled back and paused during the night shift from June to August. Although it is typical for emergency cases not to occur during the night shift, the statistical analysis may not support a conclusion regarding the effectiveness of the Beacon dispatch system during the night shift.

### 4.3. Full-Level Phase

The full-level phase aimed to experiment and evaluate the effects of the Beacon dispatch system controlling all tasks; however, it was possible that our new system could pose a risk to patients. To minimize the risk, we continued to apply our Beacon dispatch system in a limited shipment phase, as shown in the results in Table 5 and Table 6. The full-level phase only applied for a limited time from 22 November 2021 to 8 December 2021, during the nighttime shifts from 6:00 pm to 10:00 pm.

During the full-level phase, the Beacon dispatch system managed all dispatch cases; however, in some cases, the system may have failed to assign the task. If the pickup time exceeded the expected dispatch time, the task was transferred to the traditional dispatch method. As a result, approximately 77% of dispatch tasks needed to be transferred to the traditional method each day.

The data in Table 7 provide statistics for each dispatch method. Based on the results, we can infer that relying on the Beacon dispatch for all tasks can be beneficial for assigning tasks to suitable staff in the area; however, it is important to have a fail-safe method in place to transfer tasks to the traditional method when necessary.

### 4.4. Overall Results

**Figure 5:**
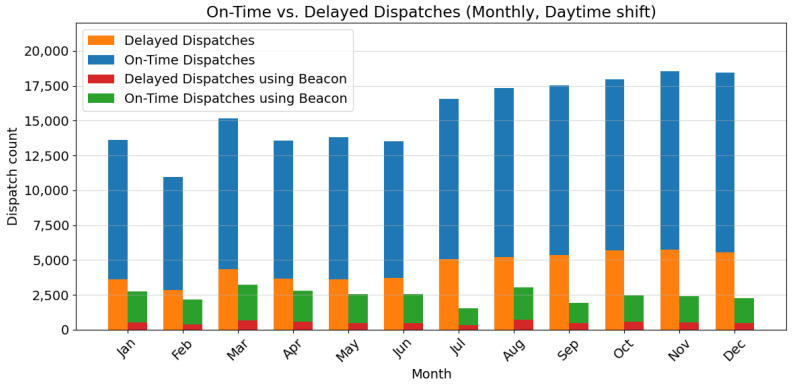
Monthly dispatch performance (daytime shift), showing the number of on-time and delayed dispatches for traditional vs. Beacon dispatch system.

**Figure 6:**
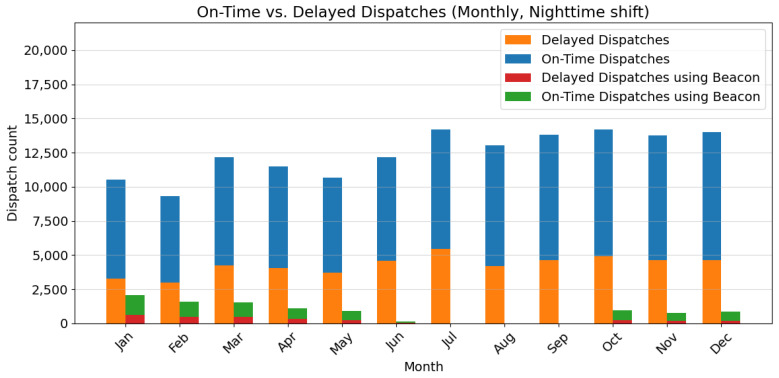
Monthly dispatch performance (nighttime shift), showing the number of on-time and delayed dispatches for traditional vs. Beacon dispatch system.

The analysis presented in Figure 5 and Figure 6 reveals key insights into the dispatch performance across day and night shifts, particularly highlighting the impact of using the Beacon dispatch system. The data indicate a consistent demand for dispatch services, with a slight increase towards the end of the year, suggesting a steady or growing demand for dispatch services. In addition, there is a significant gap between on-time and delayed dispatches, especially during the night shift, which could be attributed to the lower number of active hospital staff and patients compared to the day shift.

Notably, using a Beacon system during the day shift, when hospital activity is higher, correlates with a higher proportion of on-time dispatches. This suggests that the Beacon system may improve timeliness during daytime operations. Conversely, the impact of the Beacon system is less pronounced during the night shift, potentially due to the reduced activity levels and fewer available resources.

The findings underscore the importance of exploring strategies to reduce delays across all shifts, particularly during the night shift when resources are limited. Potential avenues for improvement include optimizing dispatch routes, streamlining procedures, and adjusting staffing levels to align with the lower activity levels during the night. Additionally, a deeper analysis of the Beacon system’s effectiveness during the night shift could yield valuable insights into maximizing its benefits during periods of lower activity.

Overall, the results suggest that the Beacon dispatch system has the potential to be a valuable asset in the future of smart hospitals, playing a crucial role in reducing delays in the dispatch process, especially during peak hours. By strategically integrating this technology and adapting its application to varying activity levels, hospitals can improve overall efficiency, provide timely responses to critical needs, and potentially reduce the number of staff needed on standby, thus optimizing labor resources.

## 5. Conclusions

This study presents a real-world implementation of a data-driven dispatch system called the Beacon dispatch system, utilizing IoT technology to optimize inpatient transportation at Taichung Veterans General Hospital. The hospital faced challenges in inpatient transportation due to budget constraints, staffing shortages, and inefficient manual processes, which often resulted in overtime delays exceeding 40% and surpassing 50% during night shifts. The Beacon dispatch system was implemented in phases, starting with a testing phase, then a limited shipment phase for urgent cases, and finally, a full-level implementation for all cases. Experimental data collected between January 2021 and December 2021 underwent statistical analysis, revealing that the Beacon dispatch system reduced overtime delays to 26.5% during daytime shifts compared to 41% using traditional dispatch methods. These findings demonstrate the transformative potential of data-driven dispatch systems in enhancing hospital operations, service quality, and patient care within smart hospitals.

In summary, the current methods of dispatching hospital patients, which rely on phone communication or staff physically returning to service centers, are inefficient and prone to delays. This negatively impacts staff workload and patient safety. Implementing a real-time tracking system using beacon technology could help address these issues by enabling faster task allocation, reducing delays, and minimizing physical strain on staff. Additionally, such a system would provide valuable data on task completion and delays, facilitating workflow optimization and improving patient safety. Our future research will investigate the impact of this technology on patient outcomes and staff satisfaction, as well as explore the potential of emerging technologies like the Artificial Intelligence of Things (AIoT).

## Figures and Tables

**Table 1:** Dispatch performance metrics: Beacon vs. traditional methods (daytime shift: January–February).

Method	Metric Name	January	February
Traditional Dispatch	Number of dispatches	9984	8118
	Number of delays	3641	2829
	Delay rate	36.5%	34.8%
	Average Dispatch Time	0:07:19	0:06:48
	Average Pickup Time	0:07:14	0:07:23
Beacon Dispatch	Number of dispatches	2226	1773
	Number of delays	515	396
	Delay rate	23.1%	22.3%
	Average Dispatch Time	0:01:18	0:01:19
	Average Pickup Time	0:04:43	0:04:33

**Table 2:** Dispatch performance metrics: Beacon vs. traditional methods (nighttime shift: January–April).

Method	Metric Name	January	February
Traditional Dispatch	Number of dispatches	7252	6332
	Number of delays	3280	2992
	Delay rate	45.2%	47.3%
	Average Dispatch Time	0:20:48	0:21:20
	Average Pickup Time	0:10:49	0:11:53
Beacon Dispatch	Number of dispatches	1414	1077
	Number of delays	637	500
	Delay rate	45.0%	46.4%
	Average Dispatch Time	0:44:31	0:44:04
	Average Pickup Time	0:11:27	0:12:09

**Table 3:** Dispatch performance metrics: Beacon vs. traditional methods (daytime shift: March–August).

Method	Metric Name	March	April	May	June	July	August
Traditional Dispatch	Number of dispatches	10830	9902	10206	9830	11461	12144
	Number of delays	4318	3661	3599	3697	5076	5191
	Delay rate	39.9%	37.0%	35.3%	37.6%	44.3%	42.7%
	Average Dispatch Time	0:08:27	0:07:31	0:07:23	0:08:23	0:09:06	0:08:07
	Average Pickup Time	0:07:30	0:07:17	0:07:11	0:07:27	0:09:33	0:09:48
Beacon Dispatch	Number of dispatches	2586	2231	2095	2065	1179	2275
	Number of delays	664	556	481	497	350	742
	Delay rate	25.7%	24.9%	23.0%	24.1%	29.7%	32.6%
	Average Dispatch Time	0:01:37	0:01:54	0:01:21	0:01:24	0:02:39	0:01:37
	Average Pickup Time	0:04:37	0:04:20	0:04:30	0:05:02	0:07:30	0:07:08

**Table 4:** Dispatch performance metrics: Beacon vs. traditional methods (nighttime shift: March–August).

Method	Metric Name	March	April	May	June	July	August
Traditional Dispatch	Number of dispatches	7901	7412	6954	7592	8728	8812
	Number of delays	4249	4055	3726	4562	5463	4207
	Delay rate	53.8%	54.7%	53.6%	60.1%	62.6%	47.7%
	Average Dispatch Time	0:21:38	0:23:00	0:24:26	0:30:14	0:33:21	0:24:30
	Average Pickup Time	0:15:21	0:14:59	0:13:53	0:12:18	0:13:36	0:10:58
Beacon Dispatch	Number of dispatches	1088	774	693	100	2	--
	Number of delays	458	345	229	33	1	--
	Delay rate	42.1%	44.6%	33.0%	33.0%	50.0%	--
	Average Dispatch Time	0:42:27	0:55:35	1:14:03	1:25:13	1:06:47	--
	Average Pickup Time	0:13:48	0:12:33	0:10:38	0:09:10	0:08:55	--

**Table 5:** Dispatch performance metrics: Beacon vs. traditional methods (daytime shift: September–December).

Method	Metric Name	September	October	November	December
Traditional Dispatch	Number of dispatches	12170	12226	12763	12891
	Number of delays	5361	5713	5764	5536
	Delay rate	44.1%	46.7%	45.2%	42.9%
	Average Dispatch Time	0:09:00	0:10:11	0:09:38	0:08:33
	Average Pickup Time	0:10:05	0:09:21	0:09:17	0:08:34
Beacon Dispatch	Number of dispatches	1456	1901	1887	1790
	Number of delays	466	565	506	473
	Delay rate	32.0%	29.7%	26.8%	26.4%
	Average Dispatch Time	0:01:42	0:01:55	0:01:38	0:01:39
	Average Pickup Time	0:06:38	0:06:58	0:07:09	0:06:25

**Table 6:** Dispatch performance metrics: Beacon vs. traditional methods (nighttime shift: September–December).

Method	Metric Name	September	October	November	December
Traditional Dispatch	Number of dispatches	9215	9267	9092	9369
	Number of delays	4608	4924	4645	4629
	Delay rate	50.0%	53.1%	51.1%	49.4%
	Average Dispatch Time	0:24:02	0:26:13	0:22:56	0:21:03
	Average Pickup Time	0:11:40	0:12:44	0:11:54	0:13:30
Beacon Dispatch	Number of dispatches	--	705	571	631
	Number of delays	--	249	208	210
	Delay rate	--	35.3%	36.4%	33.3%
	Average Dispatch Time	--	1:22:10	1:16:30	1:24:14
	Average Pickup Time	--	0:09:07	0:08:55	0:09:16

**Table 7:** Dispatch performance metrics during full-level phase (averages daily from 22 November 2021 to 8 December 2021, from 6:00 p.m. to 10:00 p.m.).

	Traditional Dispatch	Beacon Dispatch
Average number of dispatches	202	57
Average number of delays	73	16
Average delay rate	35.73%	28.18%
Average Dispatch Time	0:19:04	0:09:45
Average Pickup Time	0:22:49	0:16:12

## Data Availability

The data presented in this study are available on request from the corresponding author due to privacy and ethical reasons.
